# Cystic sellar salivary gland-like lesions 

**DOI:** 10.5414/NP301235

**Published:** 2019-12-17

**Authors:** Bette K. Kleinschmidt-DeMasters, Marc K. Rosenblum, Janice M. Kerr, Kevin O. Lillehei

**Affiliations:** Departments of; 1 Pathology,; 2 Neurology,; 3 Neurosurgery, University of Colorado Denver, Aurora, CO,; 4 Department of Pathology, Memorial Sloan-Kettering Cancer Center, New York, NY, and; 5 Department of Endocrinology, University of Colorado Denver, Aurora, CO, USA

**Keywords:** salivary, sellar, pituitary, Erdheim, Rathke cleft cyst

## Abstract

Introduction: Cystic sellar salivary gland-like lesions (CSSLs) are exceedingly rare, with fewer than a dozen case reports. They contain amorphous colloid identical to Rathke cleft cyst contents, but the cyst wall additionally shows cohesive aggregates of benign salivary glands. We report three new examples. Materials and methods: Two cases were seen at University of Colorado Denver and one at Memorial Sloan Kettering (MSK). Molecular testing was attempted on two of three. Results: Case 1 is a 20-year-old female who presented with panhypopituitarism and was found to have a suprasellar mass that proved to be a CSSL. She received no postoperative adjuvant therapy, but recurrence of headaches and blurred vision 2 years later prompted return to medical attention. A much smaller local cyst recurrence was now accompanied by a thickened, bulbous infundibular stalk. Second resection yielded a gliotic infundibular stalk and amorphous mucin, but no residual salivary-like glands. She is without further recurrence on 6-year follow-up. Case 2 is a 29-year-old female with headache; while seen initially at a tertiary care center, diagnosis was only made after consultation at MSK. Case 3 is 68-year-old female who had originally presented with apoplexy to an outside hospital 7 years prior to surgery and diagnosis. Molecular testing was uninformative on case 1 and negative for mutations or fusions on case 3. Conclusion: Few pathologists or neuropathologists have encountered CSSLs in their practices; case 1 produced recurrence and significant infundibular stalk damage, and case 3 originally manifested apoplexy, features not previously reported.

## Introduction 

Microscopic tumors of a few, well-formed salivary acini, lined by low cuboidal epithelium, were recognized to occur in the normal pituitary gland by Erdheim in 1940 [1, 2]. These incidental heterotopic salivary tissue tumors have since been referred to as “Erdheim rests”. In an exhaustive review of 2300 pituitary autopsy specimens by Schochet et al. [3] in 1974, ~ 3% of patients’ pituitary glands contain microscopic tumors of salivary gland-like tissue, and are usually asymptomatic. Interestingly, a number of other organ sites have since also been recognized to harbor these misplaced, small, heterotopic tumors of salivary gland tissue, especially in head and neck regions [4, 5]. 

Larger, symptomatic, mass-like tumors of more numerous, yet still histologically benign, salivary gland-like tissue in the sellar region are far less common, with less than a dozen case reports in the literature [2, 6, 7, 8, 9, 10, 11, 12, 13, 14]. Indeed, the names borne by these rare larger lesions such as “rest”, “hamartoma”, or “choristoma,” even when symptomatic and requiring surgical resection, indicate that the neoplastic nature of the condition is not fully accepted. 

Although symptomatic cystic sellar salivary-gland like lesions (CSSLs) usually contain amorphous colloid identical to Rathke cleft cyst (RCC) contents, they additionally consist of cohesive mass-like tumors of benign salivary glands within the cyst wall. Interestingly, CSSL is only one of several types of salivary gland lesions that can result from these small, misplaced, heterotopic rests. Indeed, a detailed 1997 description of six cases in a paper bearing the name of “Salivary gland-like tumors of the sellar region” actually consisted of diverse tumors – not the entity of CSSLs reported in the current paper [15]. Instead, those six cases were identical to their true neoplastic counterparts found in major salivary glands of the head and neck and included one oncocytoma, two monomorphic adenomas, one pleomorphic adenoma, and one low-grade adenocarcinoma [15]. 

In the current study, we detail three new patients with symptomatic CSSLs, two of whom presented with unique clinical features, including one with clinical recurrence and 6-year follow-up after the second surgery. In addition, for the first time, mutational and fusion testing was attempted on two of the three CSSLs in which material was available. 

## Materials and methods 

The personal files of the senior authors from each institution (BKD, MKR) were searched for examples of CSSLs. These were the only CSSLs that each author had seen in their 25+ year practices at each site, indicating the rarity of the entity. Both institutions serve as tertiary care centers for neuro-oncology and pituitary disorders. In addition, informal inquiries made to neuropathologists at several other very large academic centers in North America, in an attempt to increase the numbers of cases in this report, proved futile. Namely, even at many places with large numbers of pituitary cases, CSSLs have not been readily observed. 

The clinical, neuroimaging, and endocrinological information from the two cases at UCD were obtained from the medical record by the clinicians originally involved in the patient’s care (JMK, KOL). Limited clinical history was supplied by the referring neuropathologist for the MSK case. 

Genetic testing was attempted on case 1; a tissue block was submitted to Foundation Medicine, Cambridge, MA, USA. On case 3, fusion and mutational testing was conducted at the Colorado Molecular Correlates Laboratory. Mutational analysis was performed using the ArcherDx Variant Plex Solid Tumor kit (ArcherDx, Boulder, CO, USA) with added custom primers. Libraries were sequenced on the Illumina MiSeq using the v3 kit or the Illumina Next Seq using the Mid-Output kit (Illumina, San Diego, CA, USA). Raw sequencing data was processed for mutation calls using the ArcherDx Analysis platform v5.1.2. Gene fusion analysis was performed using the ArcherDx Fusion Plex Solid Tumor kit. Libraries were sequenced on the Illumina MiSeq using the v3 kit or the Illumina Next Seq using the Mid-Output kit. Raw sequencing data was processed for fusion calls using the ArcherDx Analysis platform v4.1.1.7. A list of mutations and fusions covered by these two methods is supplied in the online Supplemental Material. Material was not available for testing on case 2. 

## Results 

### Case 1 

The patient is a 22-year-old female who was initially seen at UCD in 2011 when she was diagnosed with panhypopituitarism, diabetes insipidus, hydrocephalus, and a sellar/suprasellar mass. 

**Figure 1.**
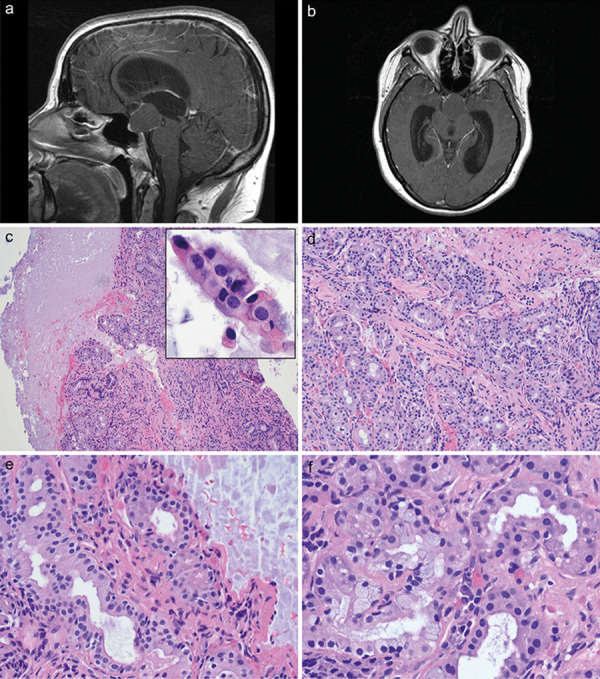
a: Sagittal T1-weighted magnetic resonance imaging (MRI) with gadolinium from case 1 demonstrates a 2.4 × 2.2 × 2.4 cm sellar/suprasellar mass with thin peripheral enhancement and associated marked ventriculomegaly/hydrocephalus of lateral and third ventricles. b: Axial T1-weighted MRI with gadolinium from case 1 illustrates the lesion caused considerable mass effect, not only resulting in enlargement of ventricles but also in the aqueduct of Sylvius. c: The surgical resection material showed a cystic sellar salivary gland-like lesion (CSSL) which consisted of a cohesive collection of small uniform glands immediately adjacent to a larger volume of acellular amorphous eosinophilic cyst contents typical of Rathke cleft cyst. The cyst contained a few small strips of low cuboidal ciliated epithelium (Figure 1c, inset). H & E, × 100, inset, × 1,000. c: The cystic sellar salivary gland-like lesion from case 1 was composed of large numbers of uniformly-sized acinar glands lined by low cuboidal epithelium without associated inflammation. H & E, × 200. e: There was no cytological atypia, mitotic activity, or necrosis in the cystic sellar salivary gland-like lesion. H & E, × 400. f: The glands focally showed goblet cells containing bubbly faintly basophilic intracytoplasmic contents as the source of mucin. H & E, × 600.

Magnetic resonance imaging (MRI) showed a large sellar/suprasellar mass measuring up to 2.4 × 2.2 × 2.4 cm, with regional mass effect as described above. There was internal heterogeneity of T2 signal within the mass and thin peripheral enhancement on T1-weighted studies with gadolinium, leading to primary considerations of a papillary type craniopharyngioma or possibly a Rathke’s cleft cyst (Figure 1a, b). The neuroradiologist felt that “the enhancement characteristics were very atypical for pituitary macroadenoma or other suprasellar masses such as pituicytoma, granular cell tumors or granulomatous hypophysitis”. 

There was moderate lateral and marked third ventriculomegaly which appeared to relate to focal stenosis of the cerebral aqueduct (Figure 1a,b). There was subtle ill-defined increased T2 signal within the tectum which was felt to relate to adhesions from the patient’s mass. Soon after admission, she underwent placement of a ventriculoperitoneal shunt and then, 10 days later, a transnasal/transsphenoidal drainage of her sellar/suprasellar cyst. 

Histology was that of CSSL, with ~ 90% of the specimen composed of acellular amorphous eosinophilic cyst contents typical of RCC (Figure 1c), with the latter containing small strips of low cuboidal ciliated epithelium (Figure 1c, inset). Approximately 10% of the material was atypical for normal RCC and consisted of a large cohesive collection of closely juxtaposed, uniformly-sized acinar glands lined by low cuboidal epithelium, and without intervening stroma or inflammation (Figure 1d). There was no cytological atypia, mitotic activity, or necrosis (Figure 1e). The glands focally showed goblet cells as the source of mucin (Figure 1f). 

Extensive post-operative evaluation by endocrinology demonstrated persistent panhypopituitarism and diabetes insipidus which had predated her surgery. She was treated with DDAVP, prednisone, estrogen/progesterone hormone replacement therapy, and Synthroid. Interval MRI scans from early-2012, mid-2012, and early-2013 showed that the original cystic lesion had resolved. 

Two years after initial surgery, she developed marked fatigue, excessive daytime somnolence, and increased generalized headaches. She also endorsed subjective blurred vision, but no peripheral vision defects. 

**Figure 2.**
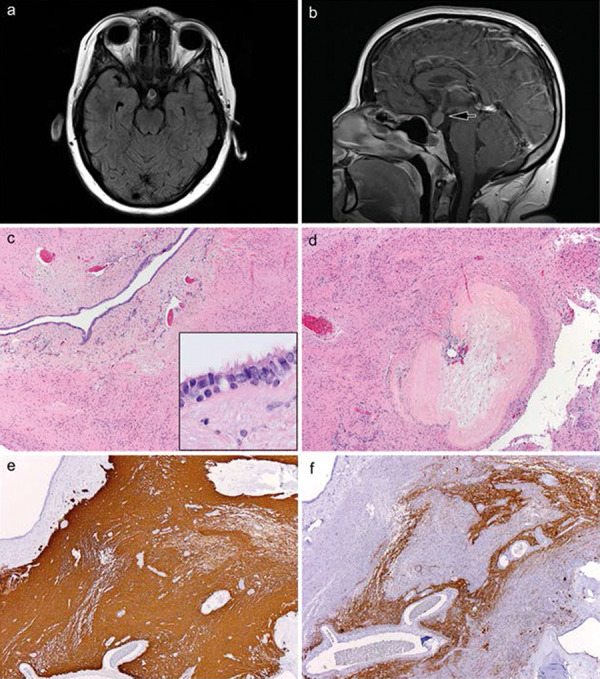
a: Axial T1-weighted MRI with gadolinium from the recurrent lesion in case 1 illustrates the recurrent cyst was now much smaller than the original lesion 2 years prior (Figure 1b), but still showed peripheral enhancement. b: Sagittal T1-weighted MRI with gadolinium from the recurrent lesion in case 1 illustrates the bulbous enlargement of the infundibular stalk and the absence of a sellar component of the recurrent cyst. c: Excisional biopsy of the stalk from the recurrent lesion in case 1 showed the central cystic component (mucinous content lost to intraoperative suctioning) within the stalk, surrounded by moderately hypercellular glial tissue. The inset illustrates the ciliated columnar epithelial lining of the cyst. H & E, × 40, inset, H & E, × 1,000. d: A nodule of dense fibrosis was also evident within the very gliotic infundibular stalk on recurrence. H & E, × 40. e: Immunostaining for glial fibrillary acidic protein (GFAP) highlighted the extremely dense gliosis in the infundibular stalk; cyst wall is seen at upper left. IHC for GFAP with light hematoxylin counterstain, × 40. f: Immunostaining for neurofilament protein (NFP) illustrates the preservation of infundibular stalk axons although with considerable splaying and distortion. IHC for NFP with light hematoxylin counterstain, × 40.

Neuroimaging studies now showed a considerably smaller cystic lesion than 2 years previously, which was confined to the pituitary stalk. The enhancing lesion along the pituitary stalk measured 7 × 14 × 6 mm; there was no intrasellar cystic component (Figure 2a). The etiology of the mass was unknown, but was “clearly not felt to represent a typical Rathke cleft cyst”. Although the previous pathology had been diagnosed as that of a CSSL, there was now concern for a coexistent craniopharyngioma due to a bulbous thickening of the infundibular stalk that had not been previously seen (Figure 2b, arrow). This had not been previously encountered in 25+ prior years of practice, at our institution, including treatment of numerous RCCs, pituitary adenomas, and 2,000+ sellar region masses. 

A right frontal craniotomy with exploration of the pituitary stalk was undertaken. Intraoperatively, the neurosurgeon could visualize the significantly widened pituitary stalk which appeared to have a grayish discoloration. The arachnoid was dissected away from both the right- and left-hand sides of the stalk, and tumor was removed in fragments. 

The tumor manifested a very firm mucinous component confined solely to the pituitary stalk, which shelled out from the internal aspect of the stalk. As the mucinous material was removed, the stalk collapsed unto itself, into a thin rim of tissue. There were noted to be striations consistent with the residual pituitary stalk, but the residual stalk was markedly thin and very abnormal. Ultimately, excisional biopsy of the stalk was performed and the lesion was felt to be “generously debulked and possibly gross totally resected”. 

Histological examination revealed 100% of the resection material to be amorphous eosinophilic colloid, this time without salivary gland-like tissue. The resection specimen of the infundibular stalk revealed that the bulbous enlargement was due to a central cyst lined by columnar ciliated epithelium (mucinous contents lost to intraoperative suctioning) surrounded by extensive, uniform gliosis on hematoxylin and eosin (Figure 2c, 2c inset). There was a large focus of collagenization as well, indicative of longstanding damage to the stalk, possibly pressure ischemia from the original very large mass (Figure 2d). Glial fibrillary acidic protein (GFAP) immunohistochemistry (IHC) verified the chronic dense gliosis and showed no cystic or rarified areas (i.e., no features of pilocytic astrocytoma with compact and loose tissues (Figure 2e)). Axons of the stalk were severely splayed apart and distorted, but generally preserved on neurofilament IHC (Figure 2f). No cytological atypia, mitotic activity, calcifications, Rosenthal fibers, eosinophilic granular bodies, or inflammation was present. Nuclei of the glial cells were devoid of elongate piloid morphology and there were only rare cells that labeled with MIB-1 IHC. Nuclear TTF-1 (thyroid transcription factor-1) was found, as is typical of infundibular stalk/posterior pituitary pituicytes. This positive immunostaining further ruled out pilocytic astrocytoma. The uniform strong immunostaining for GFAP and preservation of the interspersed axons completely excluded pituicytoma. IDH1 R132H IHC was negative and no neoplastic ganglion cells were seen on H & E or synaptophysin immunostaining. No wet keratin, calcification, or craniopharyngioma were present. Karyotype of the infundibular stalk tissue was normal 46, XX and no *BRAF* mutation was detected. 

Over the ensuing 6 years, she had not had tumor/cyst recurrence and her panhypopituitarism and diabetes insipidus have been managed by endocrinology. Molecular testing on the block submitted on this case to Foundation Medicine was technically unsuccessful. 

### Case 2 

The patient is a 29-year-old woman with headache and a pituitary mass having solid and cystic components; neuroimaging studies were not available. The neuropathologist who initially encountered the lesion had noted that it was negative for the immunostaining normally expected in a pituitary adenoma, such as chromogranin and synaptophysin. Additional immunostaining for anterior pituitary hormonal markers and transcription factors (Pit-1, SF-1 or TBX19/T-PIT) was also negative. The case was sent in consultation to MSK where diagnosis of CSSL was made. 

**Figure 3.**
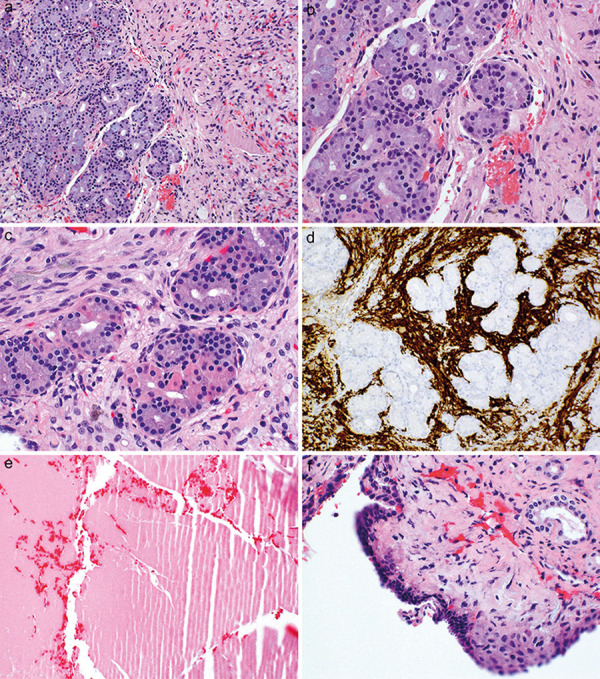
a: The excisional biopsies from case 2 were composed of closely juxtaposed, numerous benign salivary-type glands within the posterior pituitary, as seen at right. H & E, × 100. b: The cystic sellar salivary gland-like lesion in case 2 was devoid of cytological atypia or mitotic activity. H & E, × 400. c: The cystic sellar salivary gland-like lesion focally contained cells with more eosinophilic cytoplasm, possibly reflecting oncocytic change. H & E, × 400. d: The cystic sellar salivary gland-like lesion from case 2 was embedded within posterior pituitary gland, as evidenced by the negatively-immunostaining salivary-type glands surrounded by intensely positively- immunostaining posterior gland on synaptophysin (SYN). IHC for SYN with light hematoxylin counterstain, × 400. e: As is typical of most cystic sellar salivary gland-like lesions, the lesion was accompanied by abundant amorphous eosinophilic mucin identical to that seen in Rathke cleft cysts. H & E, × 100. f: Also typical of most cystic sellar salivary gland-like lesions, a cyst wall lining identical to that seen in Rathke cleft cyst was found, with pseudostratified low cuboidal lining (upper left) with possible focal squamous metaplasia (lower right). H & E, × 100.

The excisional biopsies were composed of closely juxtaposed benign salivary-type glands producing mucin (Figure 3a), without cytological atypia or mitotic activity (Figure 3b) and focally showing eosinophilic cytoplasm possibly reflecting oncocytic change (Figure 3c). The CSSL was embedded within posterior pituitary gland, as evidenced by the negatively immunostaining salivary-type glands surrounded by intensely positively immunostaining posterior gland on synaptophysin (Figure 3d). The CSSL was accompanied by amorphous eosinophilic colloid material (Figure 3e) and adjacent cyst wall lined by low cuboidal cells (Figure 3f), both identical to that of RCC (Figure 3f). 

Follow-up has been less than 1 year. 

### Case 3 

**Figure 4.**
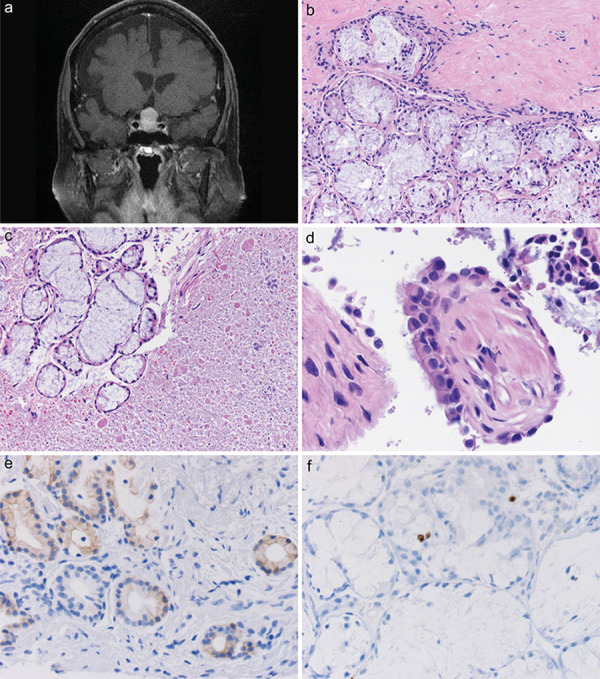
a: MRI from case 3 showed 13.5 × 14.0 × 18.6 mm T1 hyperintense mass that filled and expanded the pituitary fossa. b: Excisional biopsies from case 3 showed a large collection of closely juxtaposed, benign salivary type glands adjacent to a thin fibrotic cyst wall (top). H & E, × 200. c: Unlike the typical Rathke cleft cyst, the amorphous eosinophilic mucin comprising the cyst contents contained occasional clusters of salivary glands. H & E, × 200. d: Ciliated columnar epithelium was easily identified in the resected material on case 4. H & E, × 600. e: Faint cytoplasmic immunoreactivity for cytokeratin 20 (CK20) was found in the glands. Immunostaining for CK20 with light hematoxylin counterstain, × 400. f: Cell cycle marker MIB-1 showed rare nuclei that appeared to be from the salivary glands with labeling. Immunostaining for MIB-1 with light hematoxylin counterstain, × 400.

The patient is a 68-year-old woman whose diagnosis of symptomatic CSSL was made at UCD. She originally had presented to a physician in another state 7 years previously, with what she described as a fairly acute onset of headache and severe fatigue. She recalled a prolonged work-up that resulted in the discovery of a pituitary mass that was a presumed pituitary macroadenoma with hemorrhage/apoplexy. Full endocrinological work-up had revealed panhypopituitarism and she had been on anterior and posterior hormone replacement since then, including DDAVP (desmopressin). No surgical exploration was undertaken at that time. She subsequently developed worsening headaches, and came to UCD for evaluation and surgery. Panhypopituitarism was confirmed, and MRI revealed a 13.5 × 14.0 × 18.6 mm T1-hyperintense mass that filled and expanded the pituitary fossa and demonstrated significant suprasellar extension, resulting in mass effect on the optic nerves (Figure 4a). Surgery was performed, and intraoperatively, a large amount of what appeared to be “material consistent with mummified hematoma” was removed. The possibility of RCC material or papillary craniopharyngioma could not be excluded by the neurosurgeon (KOL). Gross total excision was felt to have been achieved. 

Excisional biopsies histologically showed a large collection of closely juxtaposed, benign salivary-type glands adjacent to a thin fibrotic cyst wall (Figure 4b); no anterior or posterior pituitary tissue surrounded these salivary gland-like tumors, and no immunostaining for transcription factors, synaptophysin or specific anterior pituitary hormones was found. The amount of amorphous eosinophilic mucin exceeded the volume of the salivary gland tumors 20-fold, but also contained occasional clusters of these salivary glands, unlike the typical RCC (Figure 4c). However, short strips of ciliated columnar epithelium identical to that expected in RCC were identified (Figure 4d). The glands were cytologically bland, showed weak cytoplasmic immunoreactivity for cytokeratin 20 (Figure 4e), and MIB-1 cell cycle labeling was seen in only rare cells that appeared to be of salivary gland origin (Figure 4f). No hemosiderin was present despite the clinical history of apoplexy 7 years earlier, but by clinical history, a second worsening of symptoms had occurred 1 year prior to surgery and final diagnosis of CSSL. Mutational and fusion testing were both negative for the mutations and fusions covered on the panels (see Supplemental Material). Follow-up time post-operatively has been less than 1 year. 

## Discussion 

CSSLs are almost always associated with a RCC component [2, 6, 10, 11], as was the case in all three of our examples. Occasionally, a RCC-like “gelatinous” cyst content is described intraoperatively and mucinous contents histologically, but cyst lining is not identified [8, 12], suggesting possible sampling error of the RCC wall component. Tanaka et al. [9] considered their CSSL to be “likely” associated with RCC, but, as in our case, did not feel that the features were typical of ordinary RCC. These six cases are the most universally cited examples of CSSLs in the literature. 

However, one of the five intrasellar “epithelial cysts” described by Fager and Carter [12] in 1966, was a symptomatic cyst associated with more abundant mucous-secreting glands than would ordinarily be seen as incidental in pituitary glands and is therefore a 7^th^ example of the entity; this case was also accepted as a CSSL by Stefanits et al. [6] and Ranucci et al. [2]. Two other reports of CSSLs have seldom been cited; these were published in Japanese [13] or Korean [14]. However, translation of these reports, and review of images suggest that, for the Korean example, “during the dura incision, the dark mucous liquid was drained” [14]. For the Japanese example [13], histopathological examination disclosed that the cyst, which was situated in the posterior lobe of the pituitary gland, “contained acid to neutral mucopolysaccharides and acinar tissue formed from simple epithelium” making these cases not only 8 and 9 on our extensive literature review, but also associated with RCC-like cyst contents. 

Finally, a related 10^th^ case of a salivary gland-type collection which was suprasellar and presented with blurred vision and hypothalamic dysfunction was intraoperatively associated with “mucinous fluid within the left optic nerve sheath” [16]. This report is clearly similar to those CSSLs cited above, albeit entitled “Intracranial salivary gland choristoma within optic nerve dural sheath: case report and review of the literature” [16]. These authors on their literature review considered cases reported by Chen et al. [10], Kato et al. [13], Kim et al. [14], and Tatter et al. [11] also to be “choristomas”. While this is a reasonable consideration, the mass-like effects of these CSSLs, especially as illustrated in our cases 1 and 3, suggests that these are abnormal growths or “masses” of salivary-like glands, even if not truly neoplastic or malignant. Unfortunately, attempts at molecular testing on our case 1, using a very broad commercially available panel, were technically unsuccessful, and no fusions or mutations were found on our case 3 by molecular testing at our own institution. 

Absence of mutation in our two cases, however, does not prove that CSSLs are not tumors, i.e., are not neoplastic. Indeed, the most commonly accepted methodology for determining if a growth is monoclonal is testing for X chromosome inactivation and involves determination of whether the same X chromosome has been inactivated within a majority of the cells in a tissue sample (referred to as non-random inactivation and highly suggestive of neoplastic rather than reactive nature of the tissue sample) [17]. X-linked markers that can be analyzed include protein isoforms, transcribed mRNAs, and the methylation status of specific gene targets, especially the human androgen receptor (*HUMARA*) gene [17]. The volume of tissue was very limited on our two samples that underwent mutational analysis (and tissue was exhausted in the process); thus this testing was not undertaken. As pointed out by Parsons [17], even if we had undertaken this testing and had found the lesions to be multiclonal, that would not have ruled out CSSL as being neoplastic, since a number of preneoplastic, benign, and malignant tumors have been found to be multiclonal. It is important to state that it is just as valid to conclude from the molecular studies that the benign salivary gland tissue may not be neoplastic. In summary, it remains unclear whether CCSLs represent true neoplasms, or not. 

Histologically, the seromucinous glands seen in CSSLs usually do not invoke diagnostic consideration of metastatic tumor, since they are cytologically bland, show perfect preservation of acinar pattern, lack mitotic activity, and are histologically analogous to incidental Erdheim rests – except for the fact that they are several-fold larger and symptom-generating along with the mucin they produce. CSSLs may be embedded within posterior pituitary tissue, as in two of our cases and the case of Kim et al. [14], or within fibrovascular tissue [2, 6, 8, 12]. The fibrovascular stroma may be identified as the cyst wall [2, 10]. Occasionally inflammation is identified [10], but was not seen in our three cases. 

As noted previously, symptomatic CSSLs are far rarer than incidental small heterotopic, non-mass producing tumors of a few salivary acini found in autopsy pituitary glands [3]. It is perhaps not surprising that rarely other types of more complex and even malignant salivary gland tumors can arise from these rests. What prompts some of these to rarely grow into more extensively mucin-producing mass lesions is unknown. The original 1997 report bearing the name “Salivary gland-like tumors of the sellar region” included no CSSLs analogous to our three cases, but instead included one pleomorphic adenoma, one monomorphic adenoma, one oncocytoma, and one low-grade adenocarcinoma [15]. Since then, at least three more reports of pleomorphic adenoma primary to the sellar region have appeared [18, 19, 20]. A case report by van Furth et al. [7] entitled “Salivary gland-like tumor of the sella” was considered a primary sellar low-grade acinic cell carcinoma by the authors themselves. Rare other primary intracranial adenoid cystic carcinomas have been reported of the right frontal lobe [21], and even in the sella [22], as well as metastases to the sella by adenoid cystic carcinomas with primary site elsewhere [23, 24, 25]. Even more frequent is invasion of the skull base by salivary gland tumors primary in head and neck regions [7, 15, 26, 27, 28, 29]. The anatomical location and clinical context allow distinction of these latter entities from sellar salivary gland-type tumors. 

Ectopic/heterotopic salivary gland tissue is not unique to the sellar region. Identical salivary tissue is not infrequently found in extracranial sites, especially in the neck area (as reviewed by Cannon et al. [30]). The embryological origin for heterotopic salivary gland tissue in the neck was noted to be possible “abnormal differentiation of local tissue, dislocation of a portion of one of the normal salivary glands, inclusion of salivary tissues in lymph nodes, and abnormal migration of normal salivary tissue” [30]. These authors discuss older literature that suggested “caudal overgrowth of the second branchial arch over the second, third, and fourth brachial clefts” for this tissue in the neck, but this embryological explanation would not be applicable to the sellar lesions in this study [30]. Interestingly, in these head and neck sites, there may be a draining sinus/fistula associated with the heterotopic tissue [14, 30]; this, however, could be analogous to the mucinous cyst contents seen in sellar examples. Sites for ectopic/heterotopic salivary gland tissue such as the rectum have also been described, which also would not fit this embryological explanation used for those in the neck [31]. Thus, the exact embryological origin of our CSSLs is unclear, but it is notable that ectopic salivary gland tissue has been found in non-sellar intracranial sites as well, including the cerebellopontine angle [32] and embedded in a solitary fibrous tumor also within the cerebellopontine angle [33]. 

The most unique aspect of the current report is the recurrence of symptoms in our case 1 and the apoplectic-like clinical presentation in case 3, neither of which has been previously reported. Case 1 had a recurrence 2 years after her initial transnasal sellar cyst drainage of a much smaller, mucin-filled cyst now confined to the pituitary stalk, and no longer within the sella. While the second surgery, including resection of the pituitary stalk, yielded only the cyst content and not the salivary-type glands, one might speculate that this component had been removed completely at the time of the first surgery and had originally been the major source of the mucinous accumulation that had yielded the original massive 2.4 × 2.2 × 2.4 cm lesion. 

In at least cases 1 and 3, the size of the mucin-filled cyst was considerable. However, with only a few cases, it would be incorrect to conclude that all CSSLs cysts are larger, or that the mucinous contents produced by the salivary type glands more abundant, than if the lesions had been only pure RCCs. 

Case 2 is less unique from a clinical standpoint, but nevertheless warrants including due to the rarity of this entity. The second case informs endocrinologists, neuroradiologists, as well as neurosurgeons, pathologists, and neuropathologists involved in the care of patients with sellar region masses that CSSLs do exist, even if they are too rare to enter into differential diagnosis. Indeed, despite the recognition of these rare lesions over 50 years ago, CSSLs are seldom encountered, even by experts. We add these three new cases to the literature and provide a literature review that places them into perspective. 

## Acknowledgment 

The authors thank Ms. Jennifer Platte for manuscript preparation, Ms. Lisa Litzenberger for photographic expertise, and Dr. Mark Ewalt from the Colorado Molecular Correlates Laboratory for mutational/fusion testing on case 3. 

## Funding 

None. 

## Conflict of interest 

The authors declare no conflict of interest. 

## Supplemental material

Supplemental material Mutations and Fusions
