# Supplementary material for: Cystic sellar salivary gland-like lesions
Source: Clin Neuropathol. 2019 Dec 17;39(3):115–25. doi: 10.5414/NP301235 (PMC8259467; doi:10.5414/NP301235)
Supplement: Supplemental material [file clinneuropathol-39-115-S01.pdf]

Mutations

(VariantPlex)

ABL1	FGFR1	MYC
AKT1	FGFR2	MYCN
ALK	FGFR3	NOTCH1
APC	FLT3	NPM1
AR	FOXL2	NRAS
ATM	GNA11	PDGFRA
AURKA	GNAQ	PIK3CA
BRAF	GNAS	PIK3R1
CCND1	H3F3A	POLE
CCNE1	HNF1A	PTEN
CDH1	HRAS	PTPN11
CDK4	IDH1	RB1
CDKN2A	IDH2	RET
CSF1R	JAK2	RHOA
CTNNB1	JAK3	ROS1
DDR2	KDR	SMAD4
EGFR	KIT	SMARCB1
ERBB2	KRAS	SMO
ERBB3	MAP2K1	SRC
ERBB4	MDM2	STK11
ESR1	MET	TERT
EZH2	MLH1	TP53
FBXW7	MPL	VHL

Fusions

(FusionPlex)

AKT3	FGR	PDGFRA
ALK	INSR	PDGFRB
ARHGAP26	MAML2	PIK3CA
AXL	MAST1	PKN1
BRAF	MAST2	PPARG
BRD3	MET	PRKCA
BRD4	MSMB	PRKCB
EGFR	MUSK	RAF1
ERG	MYB	RELA
ESR1	NOTCH1	RET
ETV1	NOTCH2	ROS1
ETV4	NRG1	RSPO2
ETV5	NTRK1	RSPO3
ETV6	NTRK2	TERT
EWSR1	NTRK3	TFE3
FGFR1	NUMBL	TFEB
FGFR2	NUTM1	THADA
FGFR3		TMPRSS2
